# Refinement of efficient encodings of movement in the dorsolateral striatum throughout learning

**DOI:** 10.1016/j.celrep.2025.116229

**Published:** 2025-09-05

**Authors:** Omar Jáidar, Eddy Albarran, Eli Nathan Albarran, Yu-Wei Wu, Jun B. Ding

**Affiliations:** 1 Department of Neurosurgery, Stanford University, Stanford, CA 94305, USA; 2 Aligning Science Across Parkinson’s (ASAP) Collaborative Research Network, Chevy Chase, MD 20815, USA; 3 Department of Neurology and Neurological Sciences, Stanford University, Stanford, CA 94305, USA; 4 The Phil & Penny Knight Initiative for Brain Resilience at the Wu Tsai Neurosciences Institute, Stanford University, Stanford, CA 94305, USA; 6 Present address: Columbia University, New York, NY, USA; 7 Present address: Institute of Molecular Biology, Academia Sinica, Taipei, Republic of China

## Abstract

The dorsal striatum plays a critical role in action selection, movement, and sensorimotor learning. While action-specific striatal ensembles have been described, the mechanisms underlying their formation and evolution during motor learning remain poorly understood. Here, we employed longitudinal two-photon Ca^2+^ imaging of dorsal striatal neurons in head-fixed mice as they learned to self-initiate locomotion. We found that both direct- and indirect-pathway spiny projection neurons (dSPNs and iSPNs, respectively) exhibited robust activation during early locomotor bouts, with activity gradually diminishing across sessions. For dSPNs, action onset and offset ensembles progressively emerged from an initially broad population of nonspecific neurons. In contrast, iSPN ensembles originated from neurons responsive to opposing actions before refining into onset- or offset-specific populations. These findings demonstrate that striatal ensemble activity becomes more selective over time, with a reduction in nonspecific neuronal activation and an increase in the efficiency of striatal encoding for learned motor actions.

## In brief

Jáidar et al. show that motor learning reshapes striatal activity by refining the tuning of its principal neuronal populations. Using longitudinal imaging, they demonstrate that dSPNs evolve from broad, nonspecific activity into distinct onset/offset groups, while iSPNs refine opposing responses into action-specific populations.

## Graphical Abstract

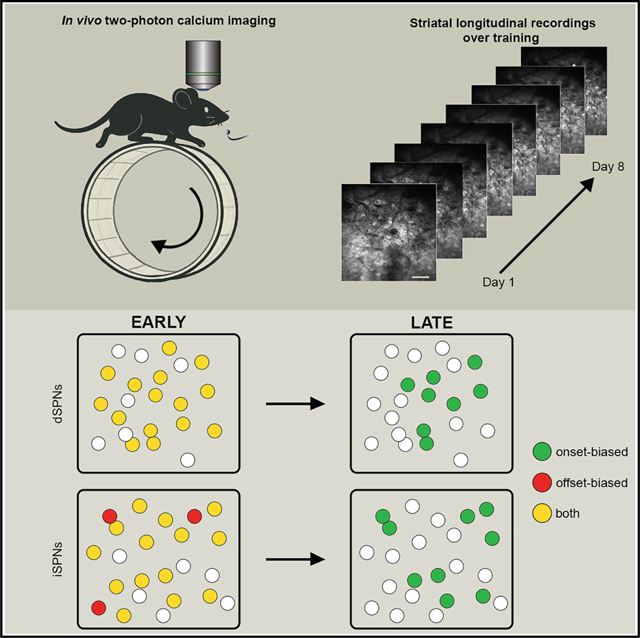

## INTRODUCTION

The nervous system possesses the remarkable ability to control animal behavior and to adapt this control to accommodate future actions and goals. This process involves the recruitment of diverse neuronal populations and circuits distributed throughout the nervous system. The striatum, the input nucleus of the basal ganglia, plays a critical role in motor control and is predominantly comprised of dopamine D1 receptors-expressing direct pathway spiny projection neurons (dSPNs) and dopamine D2 receptors-expressing indirect pathway SPNs (iSPNs).^1–4^ The striatum receives convergent inputs from various sensorimotor areas, including the cortex. Lesioning the motor cortex in animals trained to perform motor skills affects the behavior when lesions occur pre-training but not after they are well trained.^5–8^ In contrast, lesioning the striatum prevents animals from learning new motor skills^9–11^ and causes a variety of motor symptoms, including the disruption of the performance of previously learned motor skills,^8,12^ delayed movement kinematics, altered coordination, and difficulty initiating movements^13,14.^

A central question is how corticostriatal neuronal activity encodes actions and how these encodings change over time. As an animal’s behavioral repertoire expands, it is imperative that precise neuronal plasticity mechanisms are engaged that facilitate integrating novel action encodings into ongoing neuronal population activity, minimizing encoding inefficiencies and potential conflicts between different action representations. Recent studies using longitudinal imaging of neuronal activity revealed that the activity of individual neurons adapts over the course of motor learning.^15–19^ Mice learning a lever-push task leads to the formation of precise spatiotemporal activity patterns within the primary motor cortex (M1), with layer 2/3 neuron activity aligning to movement onsets and stable temporal sequences of activity emerging.^19^ In the striatum, motor learning results in neuronal activity becoming correlated with the initiation and termination of action sequences,^2^ and striatal SPNs in the dorsolateral striatum (DLS) develop reproducible firing sequences that are specific to learned movements.^20,21^ Thus, within both the M1 and the DLS, neuronal ensembles emerge during motor learning whose activations are selective to specific features of movement.

But how are neuronal ensembles formed during learning in the striatum? In particular, the specific roles played by direct and indirect pathway ensembles are not well understood. To address this question, we performed *in vivo* longitudinal two-photon imaging of striatal Ca^2+^ dynamics as head-fixed mice learned to execute voluntary bouts of locomotion on a running wheel. By comparing periods where animals initiated bouts of locomotion (onsets) to periods where they terminated locomotion (offsets), we were able to isolate two distinct action states and their corresponding striatal activity, permitting us to investigate how striatal activity encodes (i.e., predicts) these distinct actions throughout learning. As animals learned to self-locomote, we were able to study these striatal dynamics across days of learning. We found that both dSPNs and iSPNs are highly recruited during motion but that their degree of motion activation decreases throughout days. In particular, the proportion of motion-nonspecific SPNs progressively decreases throughout learning. Concurrently, we observed that SPNs are progressively refined during learning such that by the late stage of training, the action-responsive (onsets vs. offsets) ensemble sizes are smaller. Fate and regression analyses suggest that action-specific neurons do not emerge throughout learning but are instead present at the beginning of training. Furthermore, although the number of striatal neurons active during bouts of motion decreases throughout learning, their information content is preserved. In combination, these results suggest that learning coincides with a refinement of nonspecific SPNs, yielding action-specific ensembles that more efficiently encode the new actions.

## RESULTS

### Longitudinal imaging of striatal Ca^2+^ dynamics during self-generated locomotion

**Figure 1.**
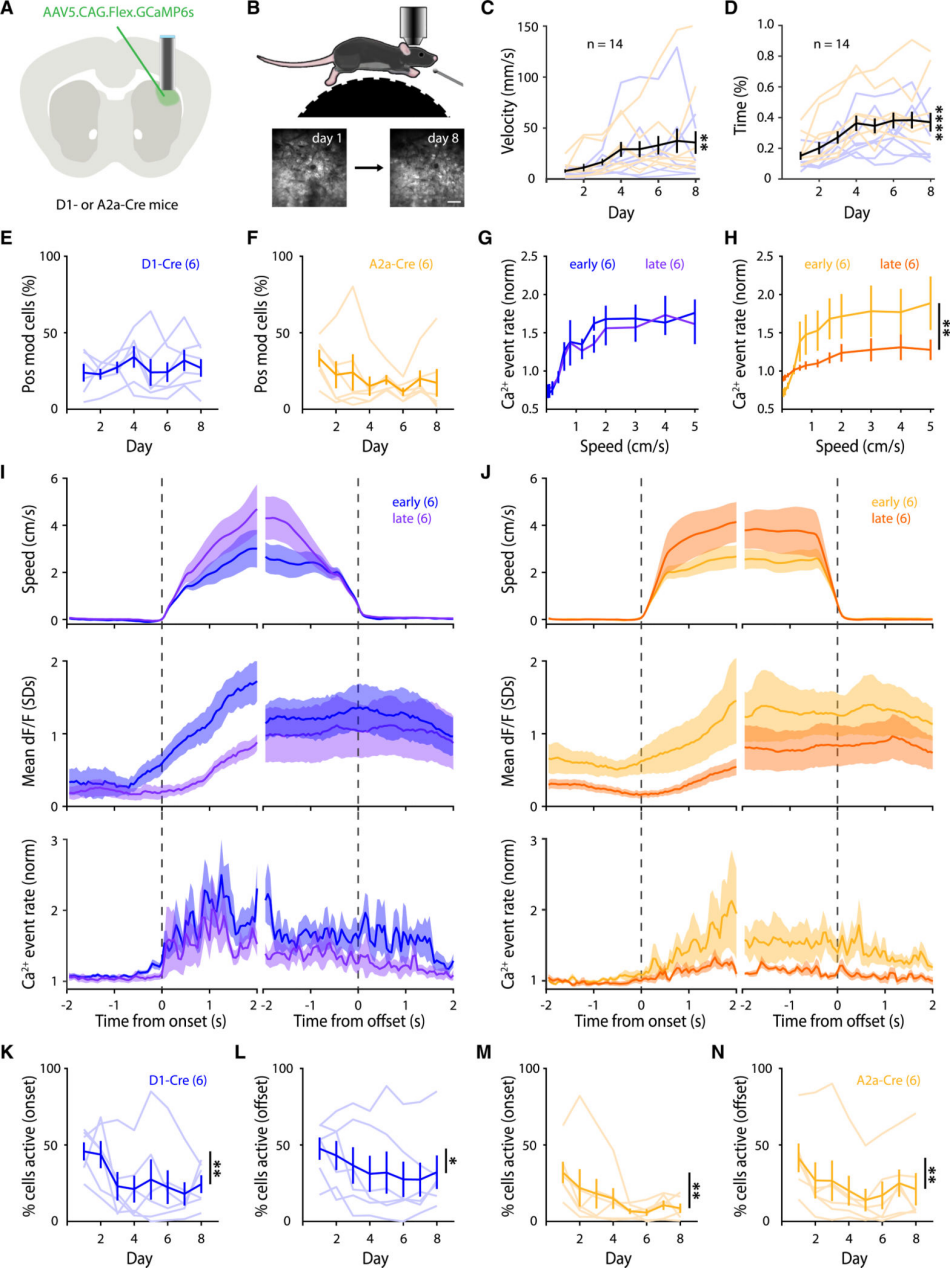
Striatal activation decreases throughout learning (A) AAV delivery of GCaMP6s into the DLS of D1-Cre or A2a-Cre mice followed by gradient refractive index (GRIN) lens implantation. (B) Two-photon (2P) imaging during head-restrained, self-generated locomotion. Bottom: DLS longitudinal imaging; scale bar: 100 μm. (C) Average velocity (*n* = 14 mice; *p* = 0.0068). (D) Average time spent running (*n* = 14 mice; *p* < 0.0001). (E and F) Percentage of imaged neurons positively modulated during locomotion for dSPNs (E; *n* = 6 mice; *p* = 0.440) and iSPNs (F; *n* = 6 mice; *p* = 0.130). (G and H) SPN event rate as a function of locomotion speed for dSPNs (G; *n* = 6 mice; *p* = 0.979) and iSPNs (H; *n* = 6 mice; *p* = 0.003). (I and J) Left: dSPN summary. Right: iSPN summary. Top: average speed. Middle: average standardized dF/F of SPNs. Bottom: average Ca^2+^ event rate (normalized). All analyses are centered around motion onsets and offsets. (K–N) Across-day percentages of dSPNs active during onsets (K, *n* = 6 mice; *p* = 0.004), offsets (L, *n* = 6 mice; *p* = 0.038), iSPNs active during onsets (M, *n* = 6 mice; *p* = 0.008), and offsets (N, *n* = 6 mice; *p* = 0.008). Statistical significance was assessed by repeated measures (RM) one-way ANOVA with multiple comparisons (C–F and K–N) and two-way RM ANOVA with multiple comparisons (G and H).

The choice of behavioral paradigm dramatically impacts the identity and dynamics of the underlying neuronal activity being studied.^22,23^ This is particularly true for the striatum, where lesions affect not only sensorimotor function but also learning and memory of various goal-directed behaviors.^24–27^ With the aim of isolating the role that the DLS plays in encoding specific motor actions, instead of training mice on a motor task with complex reward and temporal contingencies, we placed head-fixed mice on freely moving running wheels, allowing them to execute bouts of self-generated locomotion in a novel context. To longitudinally image the same SPN populations across days, an adeno-associated virus (AAV) encoding Cre-dependent GCaMP6s was injected unilaterally into the DLS of DR1a-Cre or Adora2a-Cre (A2a-Cre) mice to label dSPNs and iSPNs, respectively (Figures 1A and 1B; Video S1). Mice have a natural tendency to locomote,^28,29^ and our behavioral paradigm was designed so that the animals could freely learn to locomote in the novel head-fixed running wheel context. Indeed, across imaging sessions, mice progressively learned to locomote, as measured by the wheel’s average per-session velocity across days, as well as the proportion of session time spent running across days (Figures 1C and 1D). Tracking the fine kinematics of limb trajectories during running, we observed an increase across days in the correlation of limb trajectories between bouts, further reflecting a motor learning process (Figure S1). Importantly, our behavioral paradigm does not rely on rewards or task structure, as mice were allowed to freely execute self-generated bouts of locomotion without sensory cues or feedback based on a task structure, permitting us to investigate the relationship more directly between striatal activity and the motor actions (i.e., motion onsets and offsets) being executed. With this approach, we were able to simultaneously measure bouts of locomotion and the Ca^2+^ dynamics of ∼160 striatal neurons in each animal across days as they learned to locomote freely on the wheel (Figures S2 and S3).

### Striatal activity progressively decreases throughout learning

We first analyzed the population-level activity of dSPNs and iSPNs across days, as previous reports have described dynamic changes in average neuronal activity in the DLS as animals learn new motor skills.^12,22,30^ Consistent with the notion that both the direct and indirect pathways of the basal ganglia contribute to motion,^31,32^ we observed that both dSPNs (23.3%) and iSPNs (27.9%) were positively modulated during locomotion (Figures 1E–1H).

Similar proportions of dSPNs and iSPNs were positively modulated during bouts of locomotion on the first session (Figures 1E and 1F). However, when we quantified the relationship between locomotion speed and Ca^2+^ event rates, we observed that dSPN activation was grossly similar between early (day 1 + day 2) and late (day 7 + day 8) stages of learning (Figure 1G), whereas iSPN activation significantly decreased by the late stage (Figures 1H and S4).

Although the proportion of dSPNs being positively modulated remained stable across days, we asked if the magnitude of their activity during locomotion changed. We observed a significant decrease across days in the magnitude of locomotion-driven Ca^2+^ activation of both dSPNs and iSPNs (quantified as both dF/F and Ca^2+^ event rate; Figures 1I and 1J). Restricting our analysis to the beginning (onsets) or the end (offsets) of motion bouts yielded similar results, as both dSPNs and iSPNs exhibited decreased activation during both onsets and offsets of motion (Figures 1K–1N). Together, these results show that population-level neuronal activation in the dorsal striatum in both the direct and indirect pathways progressively decreases as animals learn to self-generate locomotion on the running wheel.

### Action initiation recruits fewer SPNs across consecutive bouts of motion

With the observed across-day changes in SPN recruitment, we next asked if we could observe dynamic changes in SPN activity across consecutive bouts of locomotion within sessions belonging to the same day of training. When we quantified the proportion of dSPNs and iSPNs active during each of the first 8 consecutive locomotion bouts (Figure S5), we observed that dSPN (Figures S5A and S5B) and iSPN (Figures S5E and S5F) activation decreased across consecutive bouts of action initiation. This effect was not observed when looking at the first 8 consecutive offsets in either dSPNs (Figures S5C and S5D) or iSPNs (Figures S5G and S5H). Together, these results suggest that initial bouts of action initiation recruit larger populations of dSPNs and iSPNs compared to later bouts, where fewer striatal cells are engaged, even within the same session.

### Decreased activation of movement-nonspecific SPNs

Striatal neurons have been shown to be functionally heterogeneous, with individual SPNs displaying activity tuned to precise action motifs, temporal features, and task structures.^8,14,27,33^ We next asked if the significant decrease in striatal activity during learned locomotion was due to a global reduction in SPN activity or if a decrease in a specific SPN population activity drove the effect. To this aim, we categorized dSPNs and iSPNs by the period of motion in which they were significantly active across a given session (onset only, offset only, both, or neither; Figure S6). These classifications were made daily based on the average activity of each individual SPN across all bouts in that day’s session (see STAR Methods).

**Figure 2.**
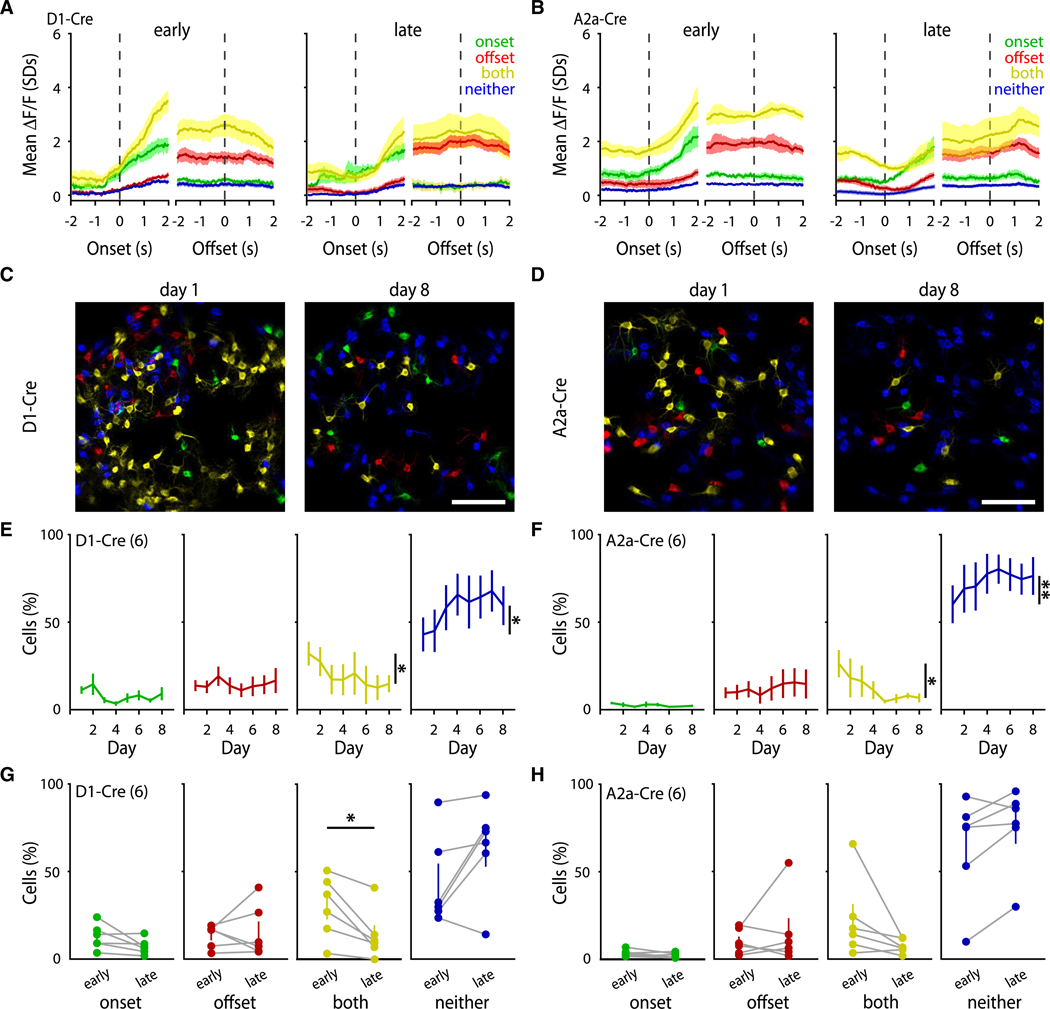
Specific decrease in the proportion of movement-nonspecific SPNs with learning (A and B) Average standardized dF/F of dSPNs (A) and iSPNs (B) SPNs are categorized as active only during motion onsets (onset only, green), offsets (offset only, red), active during both onsets and offsets (both, yellow), or not active during either (neither, blue). (C and D) Representative images of SPN activation types during sessions 1 (left) and 8 (right). Scale bars: 100 μm. (E) Percentage of dSPNs across days categorized as onset only (green; *n* = 6 mice; *p* = 0.189), offset only (red; *n* = 6 mice; *p* = 0.814), both (yellow; *n* = 6 mice; *p* = 0.027), and neither (blue; *n* = 6 mice; *p* = 0.018). (F) Percentage of iSPNs across days categorized as onset only (green; *n* = 6 mice; *p* = 0.525), offset only (red; *n* = 6 mice; *p* = 0.660), both (yellow; *n* = 6; *p* = 0.029), and neither (blue; *n* = 6 mice; *p* = 0.003). (G) Average dSPN activation types for early vs. late periods (onset only *p* = 0.102; offset only *p* = 0.710; both *p* = 0.018; neither *p* = 0.084). (H) Average iSPN activation types for early vs. late periods (onset only *p* = 0.176; offset only *p* = 0.468; both *p* = 0.064; neither *p* = 0.063). Statistical significance was assessed by RM one-way ANOVA with multiple comparisons (E and F) and two-sided paired t tests (G and H).

Categorizing dSPNs and iSPNs into these functional classes revealed that despite the decreased number of SPNs recruited during locomotion, the average activity profiles of the remaining active SPN types were grossly unchanged (Figures 2A and 2B). When we compared the proportion of functional types across early and late stages of learning, we observed that the number of onset-only and offset-only SPNs remained stable across days (Figures 2C–2F). However, the number of dSPNs and iSPNs that activated during both motion onset and offset (i.e., both) significantly decreased across sessions (Figures 2C–2H). Consequently, this decrease in both SPNs was mirrored by an increase in the proportion of SPNs that did not activate during motion onset or offset (i.e., neither; Figures 2E–2H). Together, these results suggest that the decreased striatal activity across learning is specific to the populations of dSPNs and iSPNs that grossly encode motion, leaving intact populations that are more specific to a precise action type, such as initiating or terminating locomotion.

### SPN motion-specific ensembles are formed from early active populations

**Figure 3.**
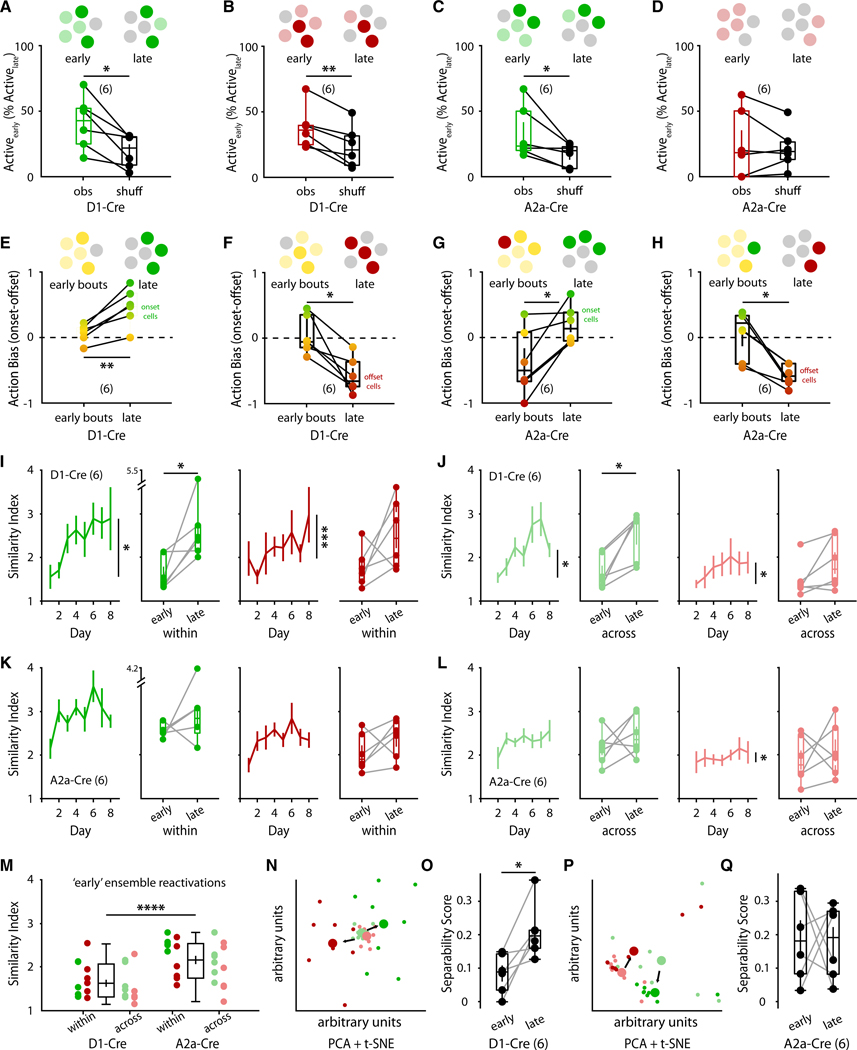
Refinement of movement-specific SPN ensembles throughout learning (A and B) Percentage of late-stage (days 7 + 8) onset (A) and offset (B) dSPN ensembles that were significantly active during early stages (days 1 + 2). Left: observed activation overlap (obs), right: average overlap of shuffled activation vectors (see STAR Methods) (*n* = 6 animals; dSPN onset: *p* = 0.017; dSPN offset: *p* = 0.003). (C and D) Percentage of late-stage (days 7 + 8) onset (C) and offset (D) iSPN ensembles that were significantly active during early stages (days 1 + 2). Left: observed activation overlap (obs), right: average overlap of shuffled activation vectors (see STAR Methods) (*n* = 6 animals; iSPN onset: *p* = 0.047; iSPN offset: *p* = 0.582). (E and F) Early vs. late activation bias averaged for each animal (across all dSPNs) for onset-specific (E; *n* = 6 animals; *p* = 0.004) and offset-specific (F; *n* = 6 animals; *p* = 0.010) dSPN ensembles. (G and H) Early vs. late activation bias averaged for each animal (across all iSPNs) for onset-specific (G; *n* = 6 animals; *p* = 0.019) and offset-specific (H; *n* = 6 animals; *p* = 0.011) iSPN ensembles. Colors depict action bias (more positive values → onset-biased → green, negative values → offset-biased → red, closer to zero → more nonspecific → yellow, E–H). (I) Within-day similarity indices for onset-specific dSPN ensembles (green; *n* = 6 mice/8 days/459 onsets; *p* = 0.045) and offset-specific dSPN ensembles (red; *n* = 6 mice/8 days/480 offsets; *p* = 8.837e− 04). (J) Across-day similarity indices for onset-specific dSPN ensembles (green; 6 mice/8 days/209 onsets; *p* = 0.045) and offset-specific dSPN ensembles (red; *n* = 6 mice/8 days/209 offsets; *p* = 0.013). (K) Within-day similarity indices for onset-specific iSPN ensembles (green; *n* = 6 mice/8 days/546 onsets; *p* = 0.637) and offset-specific iSPN ensembles (red; *n* = 6 mice/8 days/556 offsets; *p* = 0.062). (L) Across-day similarity indices for onset-specific iSPN ensembles (green; 6 mice/8 days/210 onsets; *p* = 0.159) and offset-specific iSPN ensembles (red; *n* = 6 mice/8 days/194 offsets; *p* = 0.042). (I–L) Right: boxplots depicting summary of early (days 1 + 2) vs. late (days 7 + 8) average (per animal) similarity indices (I, *p* = 0.016; J, *p* = 0.078; K, *p* = 0.016; and L, *p* = 0.047). (M) Comparison of early similarity indices between SPN types (pooling “within” and “across” days, onset + offset ensembles from I–L) (dSPN early *n* = 24, iSPN early *n* = 24; *p* < 0.0001). (N) Representative principal-component analysis (PCA) + t-distributed stochastic neighbor embedding (tSNE) dimensionality reduction of an animal’s dSPN population activity across onsets (green) and offsets (red), with early (days 1 + 2) bouts in lighter colors and later (days 7 + 8) bouts in darker colors. (O) Separability score (onsets vs. offsets) of all D1-Cre animals comparing the early- to late-stage dSPN population separability of actions (*n* = 6 mice; *p* = 0.016). (P) Representative PCA + tSNE dimensionality reduction of an animal’s iSPN population activity across onsets (green) and offsets (red), with early (days 1 + 2) bouts in lighter colors and later (days 7 + 8) bouts in darker colors. (Q) Separability score (onsets vs. offsets) of all A2a-Cre animals comparing the early- to late-stage iSPN population separability of actions (*n* = 6 mice; *p* = 0.886). Statistical significance was assessed by two-sided paired t tests (A–H, I–L, right, O, and Q), linear mixed-effects modeling (I–L, left), and two-sided unpaired t tests (M).

Motor memories are thought to be encoded by precise ensembles of neurons.^19,20,34–36^ We observed that the number of onset-specific and offset-specific dSPNs and iSPNs remained stable over days, while movement-nonspecific activity decreased. This raised an important conceptual question: are the identities of active SPNs stable across days of learning? To address this, we leveraged our longitudinal SPN tracking and first performed a fate analysis where we searched for subsets of movement-activated SPNs during early training that persisted into late training days (as nonspecific neurons generally became disengaged). Indeed, when quantifying the percentage of late-training ensembles that were previously active during early training, we found that late-training dSPN onset and offset ensembles were also significantly motion activated at early training days (Figures 3A and 3B). Conversely, although early- and late-training iSPN ensembles overlapped for onsets (Figure 3C), we found that they did not significantly overlap for offsets (Figure 3D).

We next asked what the activation type (i.e., onset, offset, or both) was of the eventual ensembles (day 7 + day 8) during the early sessions (day 1 + day 2). We first quantified the population overlap between early-training onset-only/offset-only ensembles and late-training onset-only/offset-only ensembles and observed no significant overlap (Figure S7), suggesting that the late-training ensembles instead overlap with early-training action-nonspecific populations. Indeed, by quantifying the “action bias” of these SPNs as how activated they were during early onsets vs. early offsets, we found that the eventual dSPN onset and offset ensembles were activated early on during both actions (Figures 3E and 3F). In contrast, eventual iSPN onset ensembles had an early, slight offset bias (Figure 3G), and eventual iSPN offset ensembles had significant early onset bias in activation (Figure 3H). In combination, these results suggest that late-stage dSPN ensembles are not formed from early-stage populations of the same activation class but are instead derived from early action-nonspecific dSPNs, while iSPN ensembles are formed from a mix of action-nonspecific iSPNs and iSPNs that show a very early bias to the opposing action.

### Movement-specific SPN ensembles are refined throughout learning

To further characterize the stability of movement-specific SPN ensembles throughout learning, we measured the similarity of SPN neurons reactivated across bouts of onsets and offsets. Strikingly, we observed that the bout-to-bout dSPN populations activated during motion onsets or offsets significantly increased in similarity throughout days of learning (Figure 3I). This effect held true when comparing the similarity scores of dSPN onset/offset ensembles within the same day or of ensembles across adjacent days (Figure 3J). These results suggest that the direct pathway progressively refines motion-activated dSPNs into stable populations that are to be activated during specific types of actions.

Surprisingly, iSPN populations did not undergo a significant increase in within-day or across-day similarities during onsets or offsets (Figures 3K and 3L). Instead, iSPN similarities during the first 2 training days already started significantly higher than those of dSPNs (Figure 3M). Thus, whereas the direct pathway progressively decreases its activation over days while simultaneously refining motion-specific dSPN ensembles, the indirect pathway also decreases its activation over days, but the active iSPNs already start off more consistently reactivated across bouts of motion. Consistent with this notion, when we used dimensionality reduction to compare ensemble activations between early and late bouts of motion, we found that low-dimensional dSPN encodings of onsets and offsets became progressively more separable (Figures 3N and 3O), whereas iSPN encodings did not (Figures 3P and 3Q). Together, these results suggest that the dorsal striatum refines populations of striatal SPNs engaged during bouts of motion onset or offset and that these ensembles are significantly stable and reactivated in late stages.

### Striatal movement-specific activity becomes more efficient throughout learning

**Figure 4.**
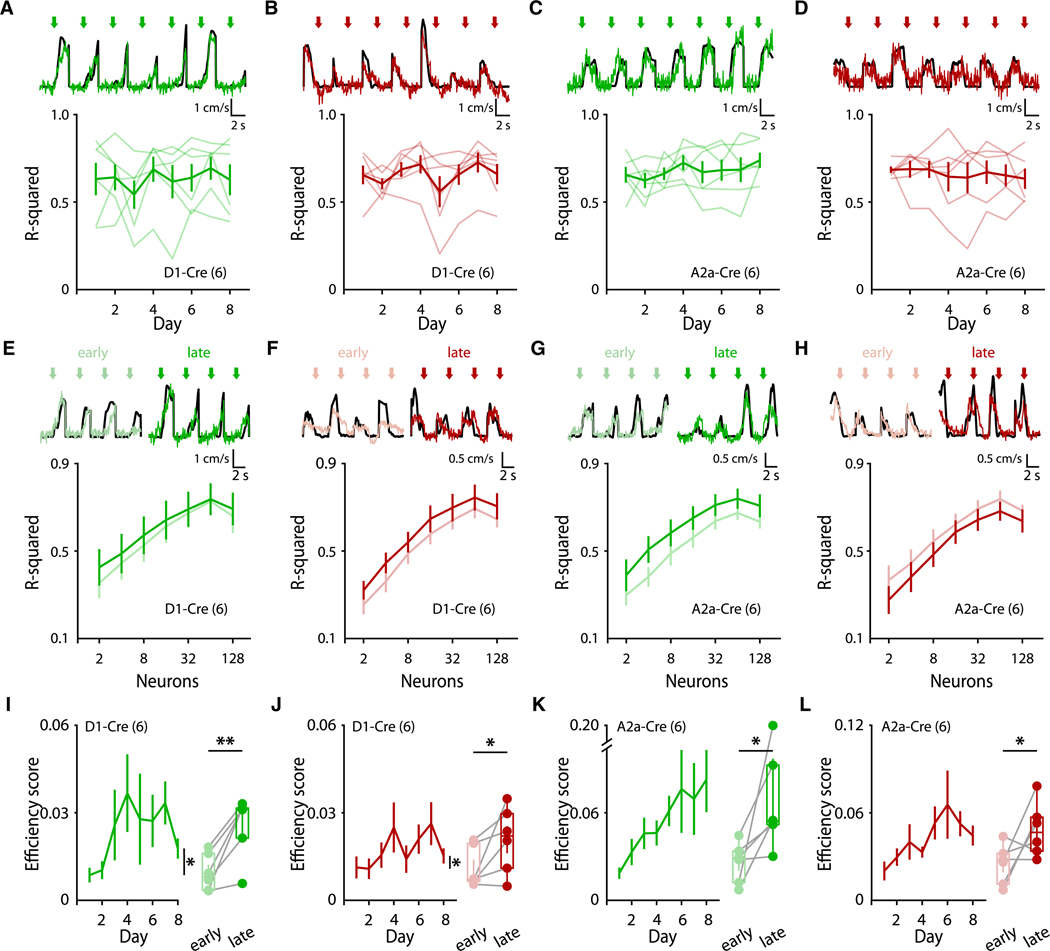
Striatal movement-specific activity becomes more efficient throughout learning (A and B) Average performance of action-specific linear regression models fitting dSPN activity to velocity, trained on subsets of onsets (A; *n* = 6 mice; *p* = 0.486) or offsets (B; *n* = 6 mice; *p* = 0.087). (C and D) Average performance of action-specific linear regression models fitting iSPN activity to velocity, trained on subsets of onsets (C; *n* = 6 mice; *p* = 0.503) or offsets (D; *n* = 6 mice; *p* = 0.814). (E and F) Model performance of action-specific (onset-specific, E; offset-specific, F) linear regression models trained on subsets of dSPNs of varying sizes (see STAR Methods; onsets: *n* = 6 mice, *p* = 0.579; offsets: *p* = 0.059). (G and H) Model performance of action-specific (onset-specific, G; offset-specific, H) linear regression models trained on subsets of iSPNs of varying sizes (see STAR Methods; onsets: *n* = 6 mice, *p* = 0.253; offsets: *p* = 0.059). (I and J) Average efficiency score (see STAR Methods) plotted for dSPN encodings of onsets (I; *n* = 6 mice; *p* = 0.015) or offsets (J; *n* = 6 mice; *p* = 0.040). (K and L) Average efficiency score (see STAR Methods) plotted for iSPN encodings of onsets (K; *n* = 6 mice; *p* = 0.076) or offsets (L; *n* = 6 mice; *p* = 0.358). (I–L) Right: comparison of early- (days 1 + 2) vs. late- (days 7 + 8) stage efficiency scores (I, *p* = 0.006; J, *p* = 0.034; K, *p* = 0.042; and L, *p* = 0.045). Statistical significance was assessed by RM one-way ANOVA with multiple comparisons (A–D), Friedman test (I–L, left), two-way RM ANOVA with multiple comparisons (E–H), and two-sided paired t tests (I–L, right).

We next asked if these dynamic changes in dSPN and iSPN activity resulted in changes in population-level encoding of movements in the striatum. To quantify the relationship between population SPN activity and their encoding of self-generated locomotion, we trained linear regression models (see STAR Methods) to predict running velocity from SPN activity. To distinguish between onset and offset encodings, we utilized separate models for motion onsets or motion offsets. When using all imaged dSPNs or iSPNs in the models, we found that model performance did not change throughout days (Figures 4A–4D), suggesting that overall information encodings in the striatum were unchanged throughout learning. Because we observed that activated SPN ensembles became more similar across training days (Figure 3), we hypothesized that late training days might engage ensembles that are more predictive of motion. However, when quantifying model performance trained on discrete subsets of SPNs, we found that model performance was not significantly changed across days, even when only including the “best” 2–8 SPNs (Figures 4E–4H; see STAR Methods). These results are consistent with our observations that motion-specific dSPN and iSPN ensembles are refined from populations of SPNs active during initial learning (Figures 3A–3H) rather than emerging as a newly activated subpopulation throughout learning.

Finally, as velocity prediction performance was unchanged throughout learning but the overall numbers of dSPNs and iSPNs active at onsets and offsets progressively decreased, we calculated the relative efficiency of striatal encoding of motion (quantified as R^2^/#SPNs). Indeed, we found that the encoding efficiency of motion onsets significantly increased throughout the training days for dSPNs and iSPNs (Figures 4I–4L). These results suggest that as dSPN and iSPN activity is refined throughout learning and motion-nonspecific activity decreases, the striatum becomes more efficient and consistent at encoding specific movements.

## DISCUSSION

Here, we characterized how striatal SPN activity encodes distinct actions and how these encodings evolve throughout motor learning. Our findings suggest that motion-specific dSPN ensembles emerge from motion-nonspecific dSPNs, which are refined throughout learning. Although iSPN ensembles did not meet significance criteria for longitudinal reactivation (Figures 3C and 3D), the high similarity of iSPN ensemble activation across movement bouts (Figures 3K and 3L) and the progressive deactivation of movement-nonspecific iSPNs (Figures 2F–2H) suggest that iSPN ensembles are also precisely defined and dynamic with learning. It is possible that the high similarity of iSPN ensemble activation at early training reflects a dynamic refinement process that occurs faster than that of dSPNs.^37,38^ At the population level, striatal activity decreases while refining these movement-specific ensembles, resulting in a more efficient encoding of movement behavior. A limitation of our study is that locomotion initiation and termination lack the kinematic richness of more traditional reaching and level-pressing paradigms, with clearer distinctions of opponent motor actions. However, a strength of our paradigm is that animals are not behaviorally shaped with reward-contingent task structures or sensory cues, each of which engages striatal dynamics, likely preventing us from isolating striatal motor encodings. Our longitudinal results provide a link between early- and late-learning SPN dynamics to elucidate how striatal ensembles are formed during learning.

The long-standing model of the basal ganglia in which dSPNs and iSPNs exert largely distinct—and often opposing—influences on motor control^3,39^ contrasts with more recent findings, where coordinated recruitment of both pathways is essential for normal action selection, execution, and learning.^29,31,32^ Our findings show that both dSPNs and iSPNs are activated during locomotor onsets and offsets, including across seemingly opposing actions (Figure 1). We observed that dSPNs are selectively reactivated and refined over the course of learning, suggesting their role in promoting specific learned actions (Figures 3A–3F), and they remain consistently engaged across bouts in later sessions (Figures 3I and 3J). In parallel, iSPNs are also robustly recruited at both onsets and offsets and demonstrate high reactivation similarity across bouts and days (Figures 3K–3M), supporting their involvement in concurrent, precisely timed modulation of movement.

Notably, our data reveal a distinct early-phase characteristic of iSPNs: action-specific ensembles initially display a bias toward the opposing action—for example, iSPNs recruited for onsets initially show stronger activation during offsets (Figure 3G). One interpretation is that iSPN ensembles are first reinforced in the context of the opposing action, consistent with their role in inhibiting actions counter to the intended movement.^40^ This might reflect a mechanism wherein iSPNs, initially active during early-onset bouts, are later recruited during offset events to facilitate transitions by suppressing onset-related activity.^40–42^ In addition to this “type reversal,” the pronounced changes we observed in iSPN motion modulation (Figure 1H) further support their dynamic role during learning.

Learning has been shown to coincide with strengthening activity between the cortex and the striatum at the cellular and synaptic levels.^11,12,43–45^ The formation of cortical ensembles has been mechanistically linked to the dynamic reorganization of synaptic inputs onto cortical cells during learning,^19,46,47^ but a role for these cortical ensembles in instructing downstream learning in the striatum has been challenging to describe mechanistically. Recent *ex vivo* findings suggest that cortical ensembles directly drive synaptic plasticity changes onto downstream SPNs, providing a possible mechanism linking cortical and striatal ensembles during learning.^48,49^

The striatum must balance the flexibility required to integrate broad inputs into SPN ensembles while simultaneously maintaining a robust stability of these memory substrates across distinct contexts.^50,51^ Our data support the notion that learning new actions involves the initial recruitment of a broad array of SPNs from which a precise ensemble is refined over repeated trials and sessions. The initial burst of SPN activation we observe in the early days (and early bouts within the same day) may reflect an intrinsic encoding flexibility that allows the striatum to generate new ensembles from pools of motion-nonspecific SPNs.^7,52,53^ Reinforcement through synaptic plasticity rules likely underlies the SPN ensemble refinement we observe, although future experiments are needed that manipulate neuromodulators, such as dopamine, acetylcholine, and the endocannabinoid system, while longitudinally recording striatal ensembles.^21,38,54–57^

### Limitations of the study

We acknowledge that, in the absence of task-specific controls, the observed striatal SPN ensemble refinements under our experimental conditions may, in part, reflect motor habituation rather than learning of a task structure.

## RESOURCE AVAILABILITY

### Lead contact

Further information and requests for resources and reagents should be directed to and will be fulfilled by the lead contact, Jun B. Ding (dingjun@stanford.edu).

### Materials availability

This study did not generate new unique reagents.

### Data and code availability

- All new data, code, protocols, and key lab materials used and generated in this study are available in the manuscript or are listed in a key resources table alongside their persistent identifiers at Zenodo: https://doi.org/10.5281/zenodo.15941678.
- Mouse behavior and Ca imaging data have been deposited at Zenodo: https://doi.org/10.5281/zenodo.11388947 and are publicly available as of the date of publication.
- Accession numbers are listed in the key resources table.
- Any additional information required to reanalyze the data reported in this paper is available from the lead contact upon request.

## STAR★METHODS

### EXPERIMENTAL MODEL AND STUDY PARTICIPANT DETAILS

#### Animals

All experiments were performed in accordance with protocols approved by the Stanford University Animal Care and Use Committee in keeping with the National Institutes of Health’s *Guide for the Care and Use of Laboratory Animals*. Animals were kept at a 12hr:12hr light/dark cycle at a room temperature of 22°C with humidity control (30–70%). For our experiments, we used heterozygous Drd1a-Cre and A2a-Cre mice. We used both male and female mice, aged ∼8 weeks at the start of behavioral experiments.

### METHOD DETAILS

#### Surgical procedures

We performed surgeries on animals under isoflurane anesthesia (1.5–2.5% in 0.5L/min of O_2_). To drive the expression of GCaMP6f in the striatum of either A2a or Drd1-Cre mice, we stereotaxically injected 400nL of AAV1-*syn*-Flex-GCaMP6f-SV40 (100833-AAV1, Addgene) into the dorsolateral striatum (from bregma, anterioposterior: 1.0mm; mediolateral: 2.25mm; and from dura, dorsoventral: − 2.45). Viral construct was injected using a pulled glass pipette at a rate of 40nL/min. After the injection, we waited for 10 min before withdrawing the pipette. We then sutured the incision and let animals recover in their cages overnight before a second surgery where we implanted an optical guide tube for GRIN lenses.

We fabricated the GRIN lenses guide tube by gluing (UV curable glue Norland #81) a 2mm-diameter #0 glass coverslip to the tip of a 3.8mm long 18G stainless steel tube (1.27mm OD, 1.06mm ID, McMaster-Carr). Excess glass was removed using a polishing wheel (Ultratec basic polisher, 30mic silicon abrasive disc).

For the implantation of GRIN lens guide tubes, we anesthetized previously injected animals with isoflurane (1.5–2% in 0.5L/min of O_2_). Using the same coordinates where the glass pipet was inserted, we generated a new craniotomy using a 1.4 mm diameter drill bit (F.S.T.). With a 27G blunt needle we aspirated the cortex until the striatum was visible (dorsoventral:−2.0mm from dura). Then the guide tube was lowered at − 2.35mm from dura. After insertion we sealed the gap between the cannula and the skull using n-butyl cyanoacrylate adhesive (Vetbond, 3M), before covering the area with dental cement (Metabon, Parkell). Finally, a second batch of dental cement was used to attach an aluminum head bar to the cranium. We allowed the animals to recover and express GCaMP6f for at least 3–4 weeks before behavioral/imaging experiments.

#### Behavioral apparatus and training

Our training device consists of a 14cm diameter microfoam wheel connected to a shaft encoder (MA3-A10–250-N, US Digital); the encoder provides a readout of the wheel revolutions and hence the travel distance and speed. Using custom code controlled by an Arduino board, we set a random reward delivery that activates a peristaltic silicon pump (Adafruit), delivering 0.2mL of water through a blunt needle at random intervals not shorter than 8 s. Random reward presentation did not engage activity dynamics of motion-driven SPNs (Figure S8). The blunt needle is connected to a capacitive touch sensor (AT42QT1010, Sparkfun) that records every time animals lick to consume the water rewards (lick-o-meter). We recorded the encoder, pump activation, and lick-o-meter signals along with the timing pulses from the microscope’s frame clock using custom made codes with a digital data acquisition system (LabView,NI). Mice underwent a water-restriction protocol whereby daily water intake was limited to ∼1mL daily. We monitored the animal’s weight daily to avoid more than a 20% drop from their starting weight and provided supplementary water as necessary. Animals training consisted of exposure to the running wheel for 8 consecutive days for ∼20 min. Mice were head-restricted with their limbs resting on the wheel, and were allowed to run at will. During this training their neuronal calcium activity was recorded using two-photon imaging.

#### Analysis of running kinematics

Videos of running sessions were recorded at 100 Hz using a Grasshopper3 camera (FLIR Systems). X-Y paw positions were then extracted using DeepLab Cut^60^ using a Linux workstation utilizing an NVIDIA GeForce RTX 2080TI graphics card. Briefly, the DeepLab Cut model was trained using ∼500 manually annotated frames, after which the model output was checked for errors and corrected. After X-Y data were extracted for each bout, the average pairwise correlation of bouts in the X and Y dimensions across all bouts for that training session was calculated.

#### Two-photon microscopy

We used a 25 ×/1.0 NA water immersion objective lens (Olympus) for striatal imaging, covering a 250× 250 μm field-of-view (FOV) through a 1 mm diameter GRIN probe #1050–002176, Inscopix). We carefully returned to the same FOV every imaging session during the 8 days of training based on the vasculature and neuronal population patters (Figure S2). A Ti:Sapphire laser (MaiTai Deepsea, Spectra-Physics) provided 920 nm excitation for two-photon imaging. We used 60–80mW laser power through the objective, as measured above the implanted GRIN probe. We detected fluorescence signals using a GaAsP photomultiplier tube (H10770PA-40, Hamamatsu) and band-pass fluorescence emission filters (FF01–520/60 for GCaMP, FF01–590/36 for tdTomato; Semrock) adapted to a custom-built two-photon microscope system with a resonant scanner (LotosScan, Suzhou Institute of Biomedical Engineering and Technology) and a 25 ×/1.0 NA water immersion objective lens (Olympus). All videos were acquired at 512 × 512 pixel resolution at ∼25 Hz frame rate. The precise temporal alignment between the Ca^2+^ imaging videos and the behavior was established by triggering the recording of all components at the beginning of each session using LotosScan.

#### Image processing and extraction of calcium signals

All recorded two-photon videos underwent the same pre-processing pipeline. First, we corrected for any DC offset in the pixel values. For each frame, we computed the minimum pixel value over the entire image. We then averaged this value over all frames and subtracted the result from all pixels in the movie. Next, we corrected for brain motion using piecewise rigid motion correction (NoRMCorre)^58^ using 32 X 32 pixel patches of each image. We then corrected for slow changes in FOV brightness throughout recordings (e.g., caused by mild bleaching of the calcium indicators). We did this by calculating the mean pixel value of the first image, fitting an exponential curve (a*exp(− b*t)+c) to the brightness as a function of each frame, and dividing each frame by its fitted brightness. Finally, we z-scored each pixel using its mean value and standard deviations over all frames.

To identify cells and extract their Ca^2+^ activity traces from the z-scored Ca^2+^ videos, we used constrained non-negative matrix factorization (CNMF)^59^ or EXTRACT^61^ cell-sorting algorithms. Candidate neuron traces were manually inspected to confirm the quality of their segmentation (spatial filters) and signal-to-noise ratio. Neurons that failed to satisfy both of these attributes were excluded from future analysis.

After extracting spatial filters corresponding to individual neurons (using CNMF or EXTRACT), we truncated the filters by setting to zero the weights of all pixels with values less than 1.5 s.d. above the mean pixel weight, averaged across the entire spatial filter. We applied these truncated spatial filters to the raw fluorescence videos to extract fluorescence traces for classified neurons. Because our data consisted of eight consecutive recording sessions, we inspected if any classified neurons of each day were present or not in other days (Figure S9). This allowed us to resolve if a cell was not detected on a given day due to a failure of the cell-sorting algorithms, or if the cell exhibited low Ca^2+^ activity. Thus, for each pair of days, we took the original spatial filters generated by the cell-sorting algorithms and computed cross-day cell matchings. Filters without any matches were seeded across days, and the Ca^2+^ activity of these candidate filters were manually inspected for clear neuronal Ca^2+^. Finally, we merged the confirmed filters for a given day, removing duplicates by generating “union” filters. Fluorescence traces were then extracted by applying the “union” filters to the z-scored Ca^2+^ videos via least-squares.

#### Detection of Ca^2+^ events and classification of active cells

Timepoints of transient activation were calculated individually for each neuron. Standardized dF/F vectors for each neuron were smoothed using a moving average with a span of 0.5 s. After calculating the derivatives of these smoothed vectors, events were identified as the peaks in the activity that exceeded a threshold of 0.4 dSD/dt. Cells were classified as active if their standardized dF/F activity exceeded a threshold of 3 SDs. Cells were further classified as Onset-only, Offset-only, of Both if they were active at onsets ±2 s, offsets ±2 s, or both at onsets and offsets, respectively. These classifications were based on each individual SPNs activity averaged across all bouts. For example, if the SPN responded sometimes to onsets and sometimes to offsets, but the averaged activity across all bouts only crosses the 3 SD threshold for the onset window and not for the offset window, it is classified as an Onset-Only SPN. These criteria are fixed for every day, across all days, to ensure consistency in classifications and confidence in any changes in activation types.

#### Classification of movement types

Motion onsets and offsets were independently determined using threshold criteria. Motion onsets were defined as timepoints preceded by a 2 s period where the rod velocity maintained below 0.09 cm/s and proceeded by a 2 s period where the rod velocity exceeded 0.35 cm/s at least once. Motion offsets were defined as timepoints preceded by a 2 s period where the rod velocity exceeded 0.30 cm/s at least once and proceeded by a 2 s period where the rod velocity maintained below 0.09 cm/s. In cases where such onsets or offsets were closer than 2 s apart, the later timepoint was omitted. Onsets and offsets were excluded if they failed the above selection criteria. For example, for a given locomotion bout (onset + offset), the onset may meet criteria (motionless before onset followed by sufficient locomotion after the onset), but the offset may fail the criteria (e.g., the animal begins a new locomotion bout too close to the offset) resulting in only the bout’s onset being used for analysis and not the offset. For motion-activation analysis, timepoints were classified as motion (motion_ts) between every motion onset and the earliest proceeding timepoint where the average velocity in a 0.7 s window fell below 0.09 cm/s. Non-motion timepoints (nonmotion_ts) were defined as all timepoints that were not motion_ts.

#### Quantifying the proportion of positively modulated cells

For each neuron, the number of active timepoints (active_ts) was calculated as the number of timepoints where its event rate was greater than 2 standard deviations. A neuron was classified as positively modulated if the proportion of active_ts/motion_ts was greater than the proportion of active_ts/nonmotion_ts. The final proportion of positively modulated cells was then determined to be the number of positively modulated cells divided by the total number of cells imaged on that day.

#### Bout to bout activation analysis

To analyze the proportion of SPNs activated across consecutive bouts (e.g., onsets), we first extracted timepoints corresponding to the first consecutive 8 onsets (or offsets), and then categorized neurons for each onset by whether or not their standardized dF/F exceeded 3 SD within a 2 s window centered at each onset (or offset). We then averaged these proportion curves across animals (per genotype) and across days in order to quantify the average bout-to-bout activation of SPNs across the first 8 consecutive bouts of any session.

#### Early-to-late ensemble reactivation fate analysis

In order to quantify the extent to which late-stage ensembles were already activated during early-stages, we compared activity-vectors across days for onsets or offsets. For each animal, we took the activation vectors for the first two days (early) or the last two days (late) and created ‘early’ and ‘late’ activation vectors by defining a neuron as active if it active on both of the two days. We then determined the amount of pre-existing activation by calculating the % of ‘late’ neurons that were also active during ‘early’ training.

#### Early-to-late ensemble type fate analysis by quantifying Action Bias

To retroactively determine the activation ‘types’ that SPNs belonged to before they became eventual action-specific ensembles, we took the activation vectors for the last two days (late) and created ‘late’ activation vectors (averaged over bouts of both days) for onsets and offsets. Averaging over the early bouts (bout 1 and 2) of early days (days 1–3) we then assigned each SPN as ‘1’ if they were on average active during onsets, ‘−1’ if active during offsets, and ‘0’ if active during both onsets and offsets (else they were omitted), yielding an ‘early bouts’ vector that we then averaged for each animal to create an overall ‘Action Bias’ for each animal.

#### Ensemble activity similarity index

To quantify the similarity between two neuron ensembles for bouts of onsets/offsets within the same day or across days, analysis was restricted only to neurons that were detectable across all 8 days of behavior. For each onset/offset bout, ensemble vectors were created such that the i^th^ value in the vector was 1 if the i^th^ neuron was active (see above) during that movement bout, otherwise the value was 0. In order to compare the similarity between two ensembles, we computed the Jaccard index: the number of shared activated neurons in the respective ensemble vectors, divided by the total number of activated neurons across both ensembles. Each Jaccard index was normalized to the average Jaccard index of 1000 shuffles of the two ensemble vectors.

#### Action Separability Score

In order to quantify how separable onset and offset actions were at the level of population Ca^2+^ activity, we first generated a matrix N consisting of rows corresponding to each onset and offset on a given day, and the columns being horizontally concatenated timeseries of each SPN imaged on that day (where the timeseries are 4 s windows centered around the onset or offset corresponding to the row). This N matrix then underwent dimensionality reduction, first using PCA (preserving 8 principal components), and then using t-SNE using the Chebyshev distance metric. Separability scores were then calculated as the [day1+ day2] average or [day7+ day8] average silhouette cluster scores for ‘early’ and ‘late’ stages respectively.

#### Peri-event regression analysis

To measure the ability of SPN Ca^2+^ activity to predict rod velocity during onsets and offsets, we first isolated 4-s windows around motion onsets or offsets, and temporally concatenated these bouts to create a singular timeseries vector of motion onsets or offsets for each day. Using the same timestamps, the Ca^2+^ activity of each SPN was concatenated to capture their activity before and after these events. We then fit linear regression models using the Ca^2+^ matrices to predict the rod onset or offset vectors. Models were k-fold cross-validated (where k was equal to the number of onsets or offsets). Using this method we used the adjusted R-squared metric to quantify the relationship between actions and population-level activity across days.

#### N-neuron regression analysis

In order to measure the amount of information encoded by subsets of SPNs of increasingly larger sizes, we trained linear regression models as described above, but this time only utilizing 2, 4, 8, 16, 32, 64, or 128 neurons. To determine which neurons to use in the N-neuron models we used LASSO regression, varying the lambda parameter to achieve a model sparsity equal to the desired N count. Once the N neurons were identified, we then fit a new linear regression model using only the N neurons using the same cross-validation procedure described above.

#### Efficiency score

To determine the efficiency of striatal encoding of actions, we used the per-neuron regression curves (see above) and the previously quantified number of active neurons per day and quantified the R-squared value corresponding to that number of neurons. Specifically, X curves were fitted to the per-neuron regression curves of each day, to match an R-squared value to a given number of activated neurons. This metric, which we the named efficiency score, reflects the amount of information encoded divided by the number of neurons activated to yield that R-squared value.

### QUANTIFICATION AND STATISTICAL ANALYSIS

Animal subjects were randomized within experimental blocks to yield equal sampling of experimental conditions. All experiments were conducted in a blind fashion (i.e., experimenter did not know the mouse genotype). Data distribution was assumed to be normal, but this was not formally tested (hence individual data points are presented in all statistical comparisons). Repeated measurements (e.g., measure of average rod velocity across days) were analyzed using 1-way or 2-way repeated measures ANOVA with post-hoc tests. All two-sample comparisons (e.g., Similarity scores) were analyzed with t-tests. Unless otherwise specified, two-sided statistical tests were conducted and data is presented as mean ± SEM (standard error of the mean), with all statistical tests, statistical significance values, and sample sizes described in the figure legends.

## Supplementary Material

1

2

4

5

6

7

8

9

10

11

Supplemental information can be found online at https://doi.org/10.1016/j.celrep.2025.116229.

## Figures and Tables

**Table:** KEY RESOURCES TABLE

REAGENT or RESOURCE	SOURCE	IDENTIFIER

Bacterial and virus strains		

AAV1-*syn*-Flex-GCaMP6f-SV40	Addgene	100833-AAV2

Deposited data		

Behavioral and Calcium Imaging raw data	Zenodo	doi:https://doi.org/10.5281/zenodo.11388947
Two-photon microscopy quantification	Zenodo	doi:https://doi.org/10.5281/zenodo.11388947
Ca2+ event timepoints	Zenodo	doi:https://doi.org/10.5281/zenodo.11388947
Early-to-late ensemble reactivation fate activity vector data	Zenodo	doi:https://doi.org/10.5281/zenodo.11388947

Experimental models: Organisms/strains		

Adora2a-Cre mice	MMRRC	RRID:MMRRC_031168-UCD
Drd1a-Cre mice	The Jackson Laboratory	RRID:IMSR_JAX:028298

Software and algorithms		

MATLAB custom made codes		
LabView	Emerson	RRID:SCR_014325
MATLAB	MathWorks	RRID:SCR_001622
NoRMCorre	Pnevmatikakis and Giovannucci^58^	doi:https://doi.org/10.1016/j.jneumeth.2017.07.031
Constrained non-negative matrix factorization (CNMF)	Pnevmatikakis et al.^59^	doi:https://doi.org/10.1016/j.neuron.2015.11.037
EXTRACT	Schnitzerlab	https://github.com/schnitzer-lab/EXTRACT-public

Other		

Protocol: Surgical prep for striatal long term imaging.	protocol.io	doi:https://doi.org/10.17504/protocols.io.kxygxy34kl8j/v1
Protocol: Behavioral training	protocol.io	doi:https://doi.org/10.17504/protocols.io.kxygxy34kl8j/v1

## Highlights

- Striatal activity evolves over motor learning
- Striatal activity reduces nonspecific signals and enhances learned-action encoding
- dSPNs shift from broad, nonspecific activation to distinct onset/offset ensembles
- iSPNs start as opposing-action responders and refine into onset/offset groups
