# Supplementary material for: Refinement of efficient encodings of movement in the dorsolateral striatum throughout learning
Source: Cell Rep. Author manuscript; Available in PMC 2025 Nov 6. (PMC12590872; doi:10.1016/j.celrep.2025.116229)
Supplement: 1 [file NIHMS2113399-supplement-1.pdf]

Cell Reports, Volume *44*

## Supplemental information

# Refinement of efficient encodings of movement in the dorsolateral striatum throughout learning

**Omar Jáidar, Eddy Albarran, Eli Nathan Albarran, Yu-Wei Wu, and Jun B. Ding**

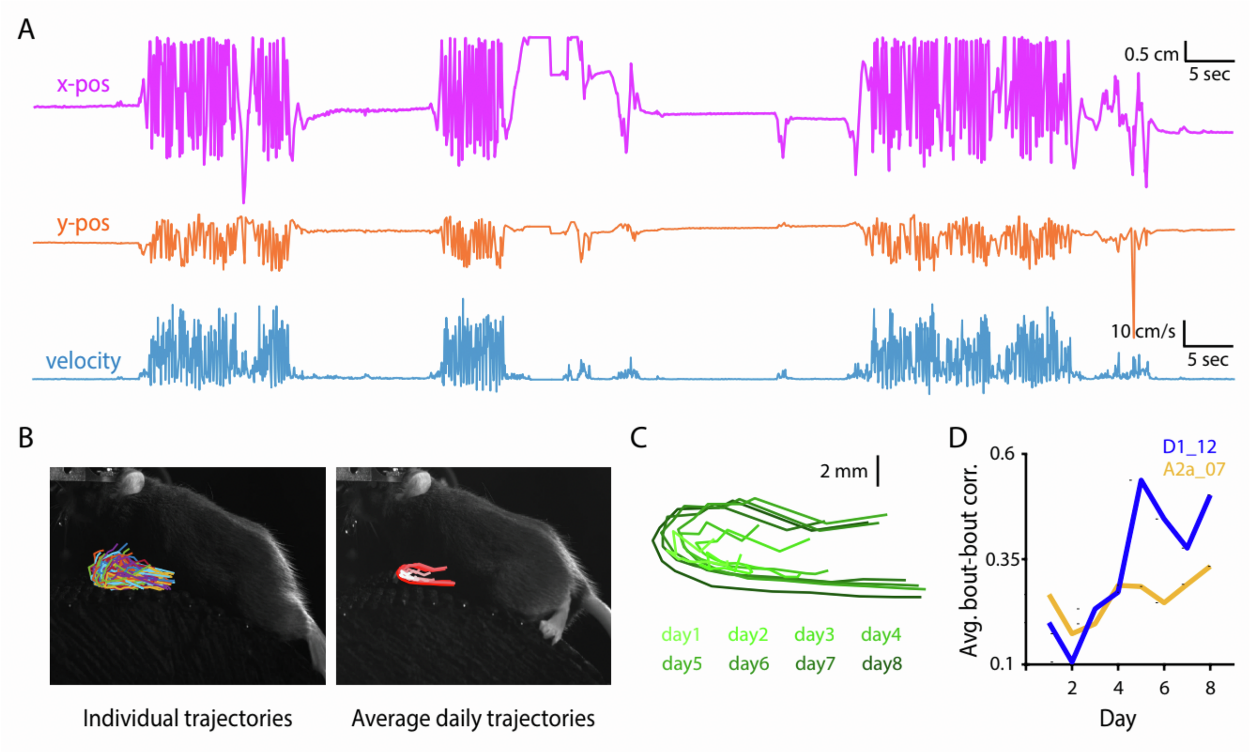


**Figure S1 – Limb kinematics become more similar across days of running.**
**(A)** Representative forelimb paw positions (x + y) and paw velocity extracted using DeepLabCut.
**(B)** Individual extracted forelimb paw trajectories overlayed for each bout (left) and averaged trajectories for each of the 8 days overlayed (right).
(**C)** Average forelimb paw trajectories across all running bouts for each of the 8 days.
(**D**) Average bout-to-bout paired trajectory correlation across days depicts increase in movement stereotypy.

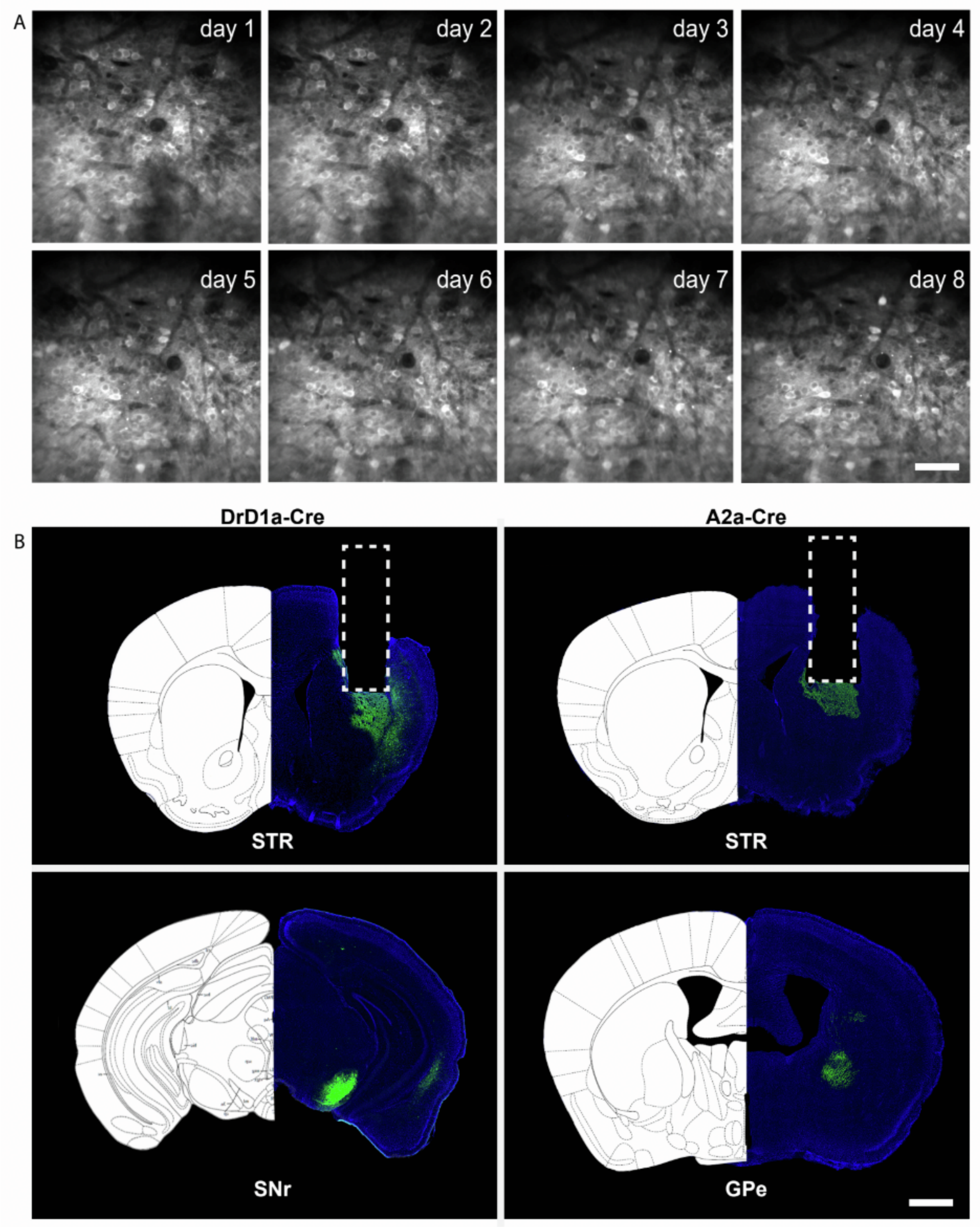


**Figure S2 – Longitudinal imaging of striatal SPNs.**

**(A)** Representative imaging of the DLS through the 2-photon microscope, depicting consistent revisiting of the same field of view across sessions. Scalebar: 100 μm.

**(B)** Representative images showing GCaMP6 expression in the DLS and their target area in DrD1a-Cre and A2a-Cre mice. Scalebar: 1 mm.

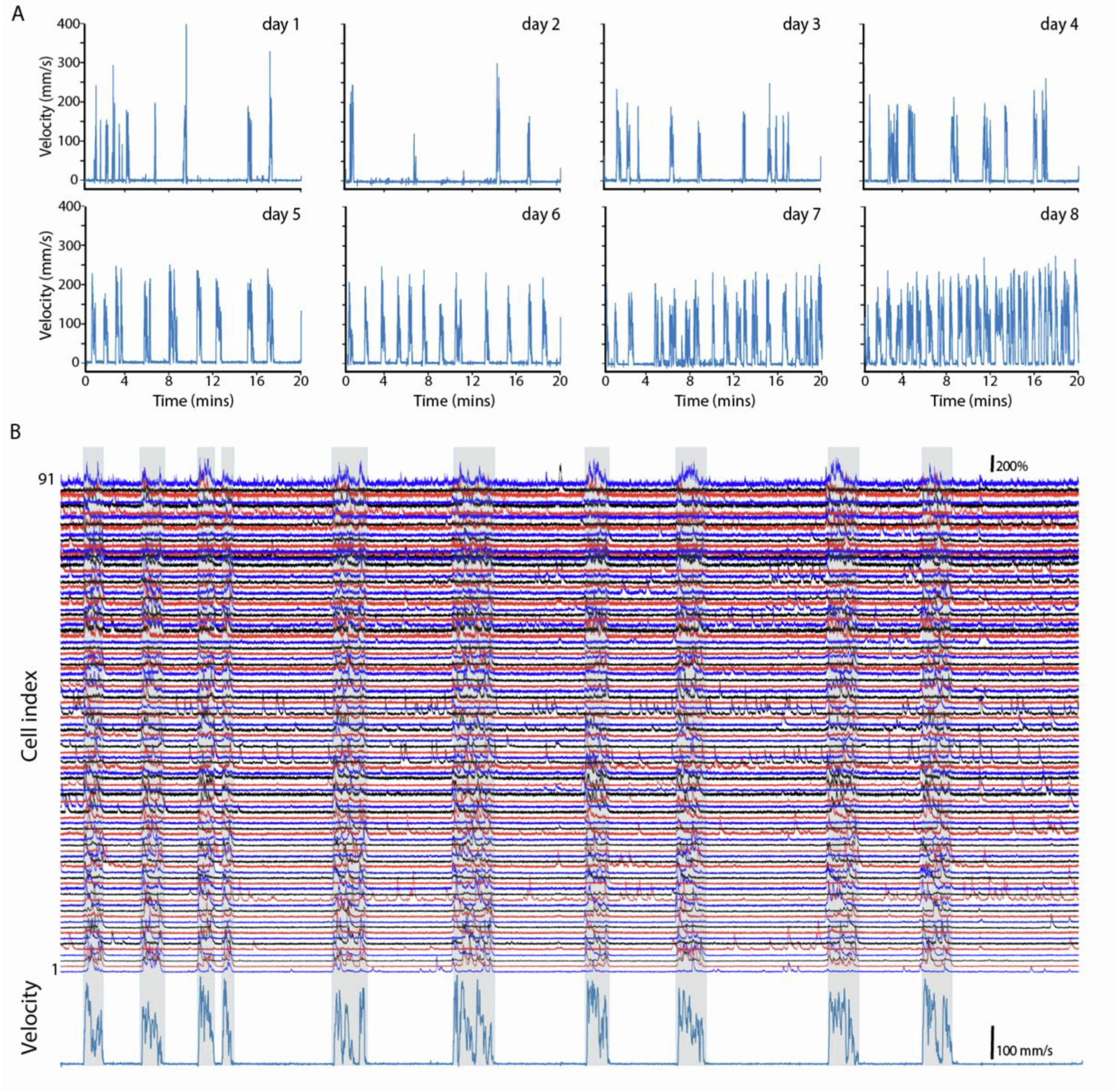


**Figure S3 – Imaging striatal SPNs throughout locomotion paradigm.**
**(A)** Representative plots of an animal's velocity across days.
**(B)** Representative $Ca^{2+}$ traces (top) of imaged SPNs as mice perform self-generated bouts of locomotion (bottom).

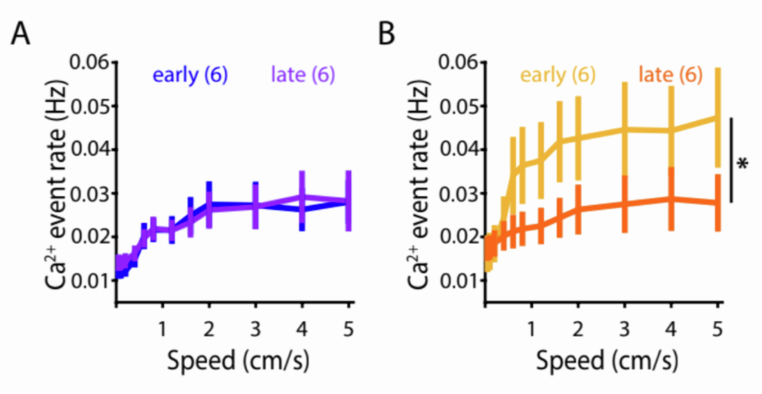


**Figure S4 – SPN $Ca^{2+}$ event rate vs. running (not normalized).**
**(A-B)** SPN activity (event rate) plotted as a function of locomotion speed, averaged across 'early' and 'late' sessions for dSPNs (**A**; n = 6 mice; p = 0.996) and iSPNs (**B**; n = 6 mice; p = 0.011).

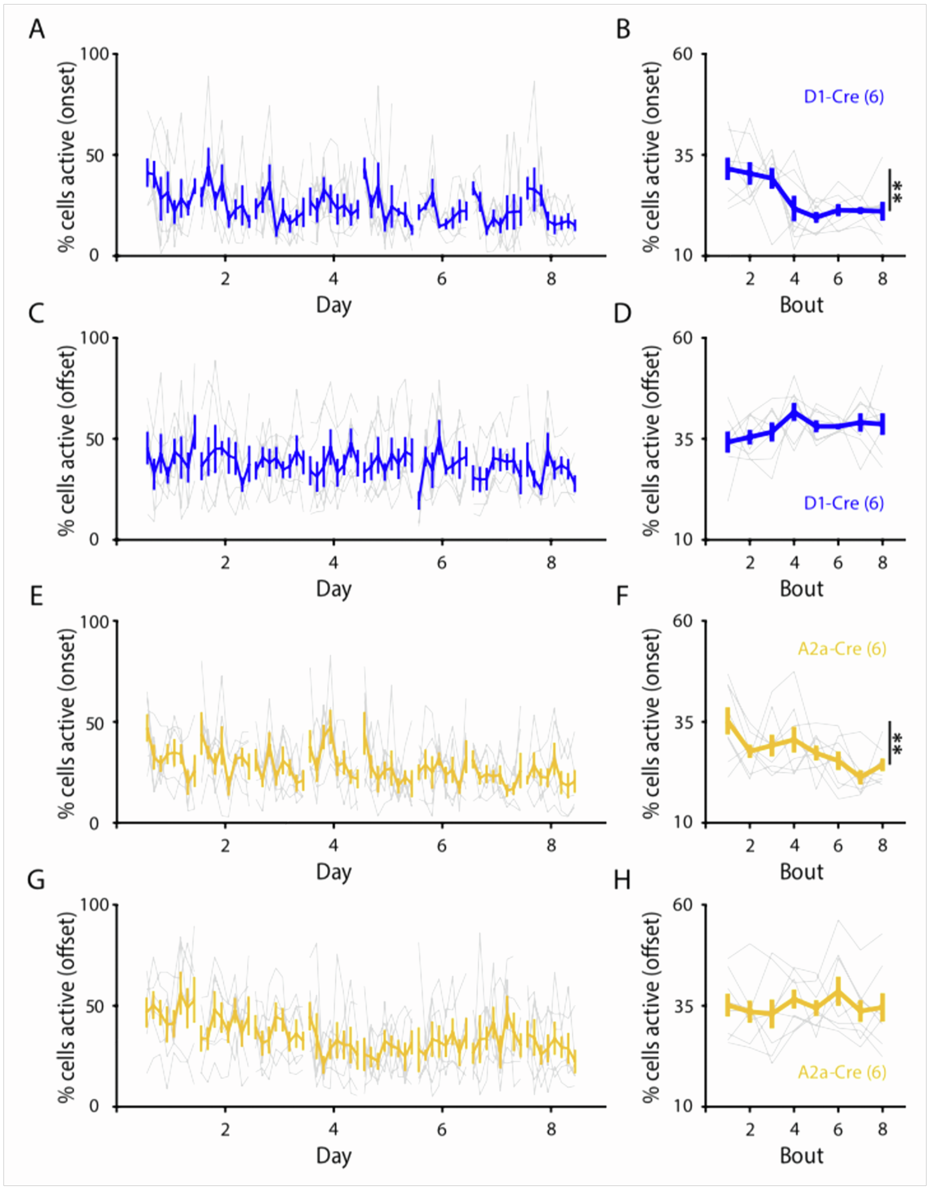


**Figure S5 – Bout-to-bout decrease in SPN activation during action initiation.**
**(A)** Percentage of dSPNs active during the first 8 motion onsets of each day (averaged across animals).
**(B)** Activation percentages for the first 8 bouts averaged across days revealing a significant decrease in dSPN activation across consecutive motion onsets (n = 6 mice; p = 0.003).
**(C-D)** dSPN activation across the initial 8 bouts of motion offsets (n = 6 mice; p = 0.340).
**(E)** Percentage of iSPNs active during the first 8 motion onsets of each day (averaged across animals).
**(F)** Activation percentages for the first 8 bouts averaged across days revealing a significant decrease in iSPN activation across consecutive motion onsets (n = 6 mice; p = 0.009).
**(G-H)** iSPN activation across the initial 8 bouts of motion offsets (n = 6 mice; p = 0.449). Data are mean ± SEM. Statistical significance was assessed by repeated measures 1-way ANOVA with multiple comparisons (**B, D, F, H**).

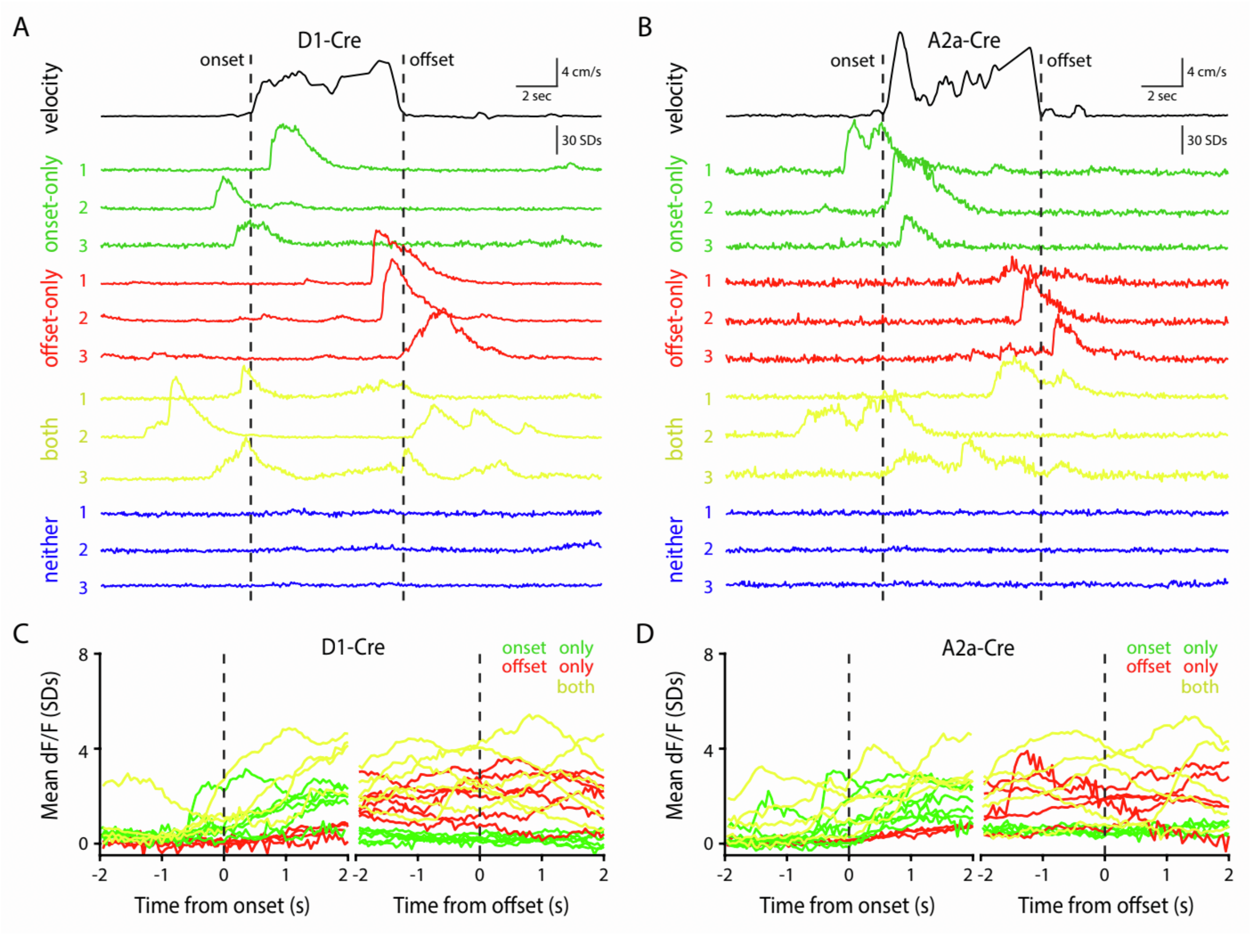


**Figure S6 – Heterogeneity in dSPN and iSPN responses during locomotion onsets and offsets.** **(A-B)** Example representative bouts of locomotion (top) in a D1-Cre animal (**A**) and an A2a-Cre animal (**B**), with 3 representative SPNs of each activation classification type ('onset-onset', 'offset-only', 'both', and 'neither) aligned to the motion onset and offset timepoints.

(**C-D**) Example representative individual dSPN (**C**) and iSPN (**D**) $Ca^{2+}$ activity centered around locomotion onset and offsets, highlighting the heterogeneity of $Ca^{2+}$ event timings relative to the onset and offset timepoints. SPN $Ca^{2+}$ activity is color-coded based on classified activation type.

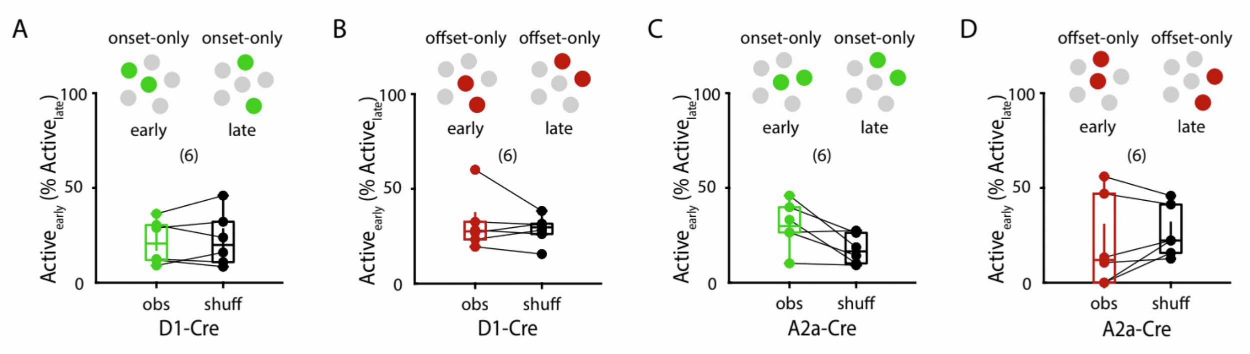


**Figure S7 – Late-training action-specific ensembles do not overlap with early-training action-specific ensembles.**

**(A-B)** Percentage of 'late' stage (days 7+8) onset (**A**) and offset (**B**) dSPN ensembles that overlap with onset-only and offset-only ensembles observed during 'early' stages (days 1+2). Left: observed activation overlap (obs), right: average overlap of 1000 shuffled activation vectors (shuff, see Methods) ($n = 6$ animals; dSPN onset: $p > 0.999$; dSPN offset: $p = 0.563$).

(**C-D)** Percentage of 'late' stage (days 7+8) onset (**C**) and offset (**D**) iSPN ensembles that overlap with onset-only and offset-only ensembles observed during 'early' stages (days 1+2). Left: observed activation overlap (obs), right: average overlap of 1000 shuffled activation vectors (shuff, see Methods) ($n = 6$ animals; iSPN onset: $p = 0.063$; iSPN offset: $p = 0.438$). Statistical significance was assessed by Wilcoxon signed rank test.

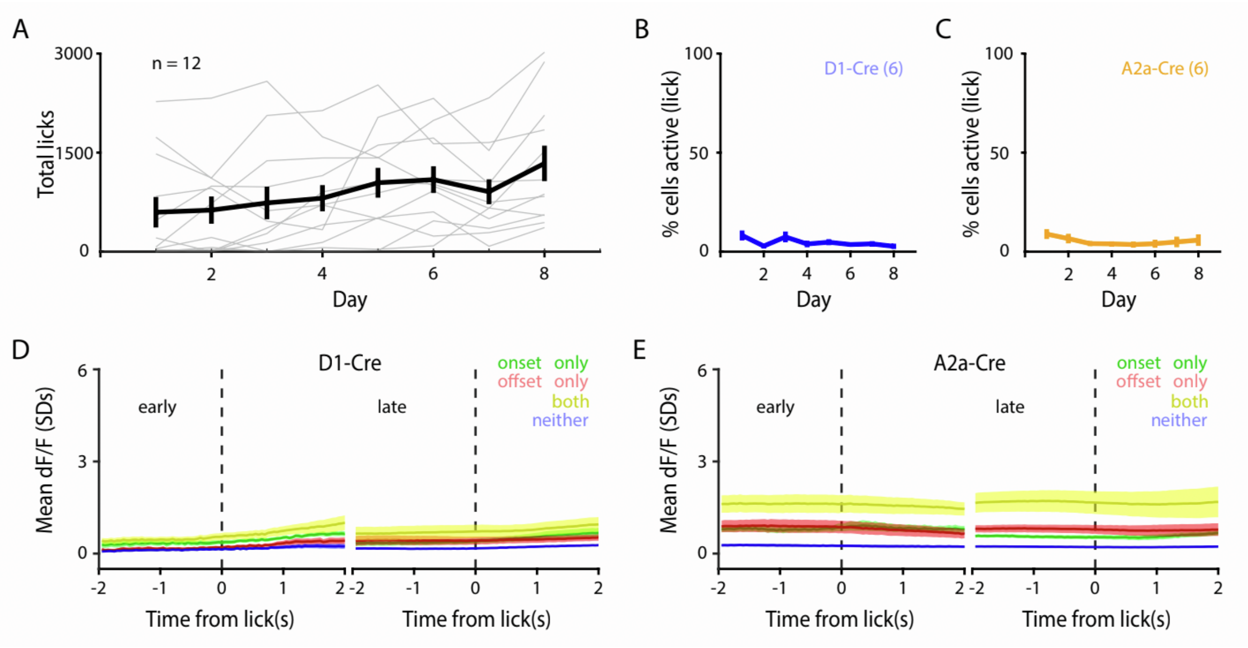


**Figure S8 – Action-responsive SPNs are not activated by reward licking.**
**(A)** Number of licks mice perform across days of imaging sessions (n = 12 mice, 6 D1-Cre + 6 A2a-Cre; p = 0.053).
**(B)** Percentage of dSPNs activated by reward licking across days (n = 6 mice, p = 0.089).
(**C**) Percentage of iSPNs activated by reward licking across days (n = 6 mice, p = 0.102).
(**D**) dSPN $Ca^{2+}$ dynamics around reward licking timepoints during early (left) and late (right) training days demonstrate that motion-activated dSPNs are not activated during reward licking.
(**E**) iSPN $Ca^{2+}$ dynamics around reward licking timepoints during early (left) and late (right) training days demonstrate that motion-activated iSPNs are not activated during reward licking. Statistical significance was assessed repeated measures 1-way ANOVA with multiple comparisons (**A-C**).

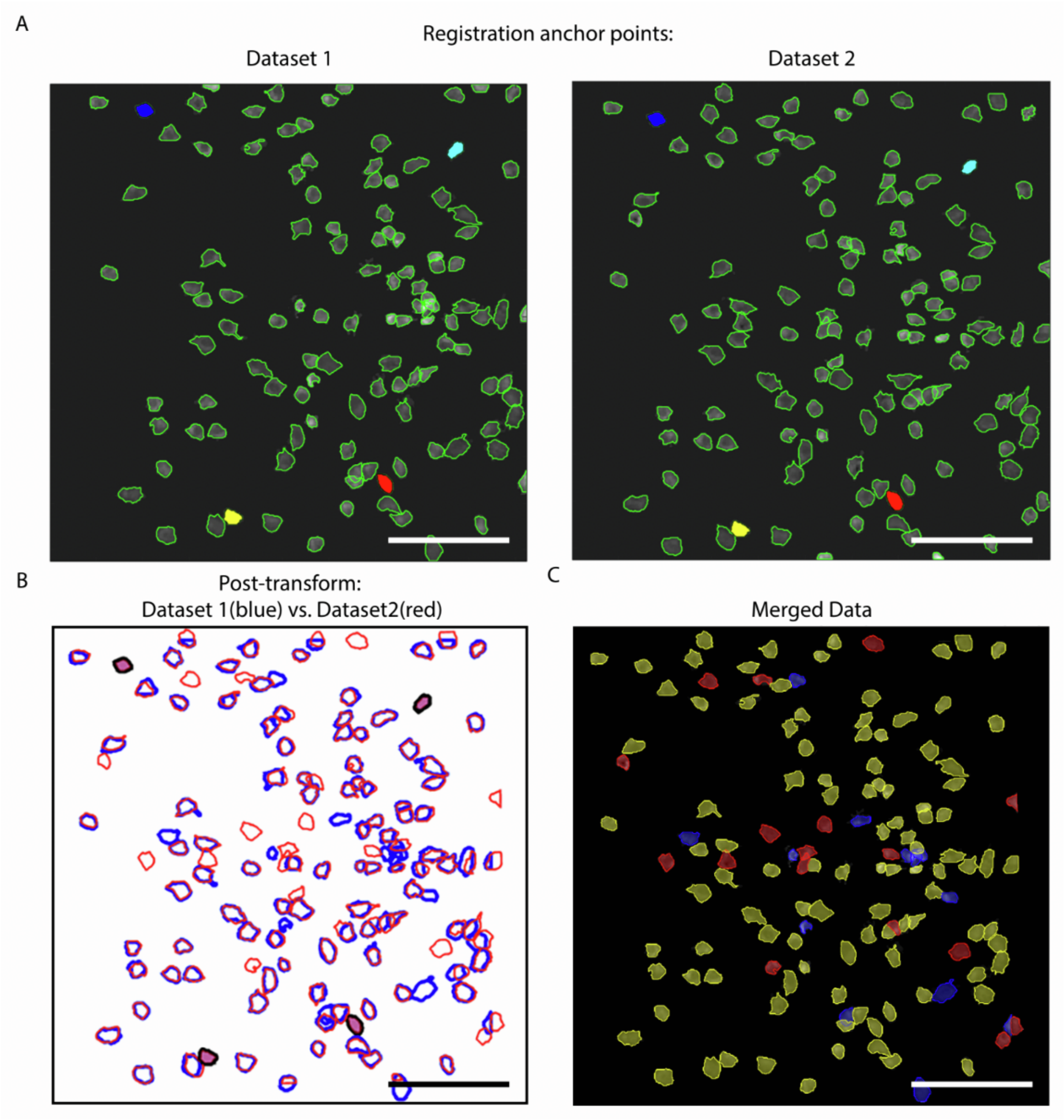


**Figure S9 – Cross-day neuronal alignment.**

**(A)** Selection of 4 pairs of active neurons shown with different colors in both data sets (dark blue, light blue, yellow, and red filters).

**(B)** Post-transformation alignment of both fields of view (FOV). Blue filter contours denote active neurons from data set 1, and red filter contours denote active neurons from data set 2. Filled filters represent the original anchor points used in **A**.

**(C)** Merged FOV image. Blue filters indicate neurons active only in data set 1, red filters indicate neurons active only in data set 2, and yellow filters represent neurons that were active in both data sets. Scale bars: 100µm.
